# Automated lung segmentation on chest MRI in children with cystic fibrosis

**DOI:** 10.3389/fmed.2024.1401473

**Published:** 2024-11-12

**Authors:** Friedemann G. Ringwald, Lena Wucherpfennig, Niclas Hagen, Jonas Mücke, Sebastian Kaletta, Monika Eichinger, Mirjam Stahl, Simon M. F. Triphan, Patricia Leutz-Schmidt, Sonja Gestewitz, Simon Y. Graeber, Hans-Ulrich Kauczor, Abdulsattar Alrajab, Jens-Peter Schenk, Olaf Sommerburg, Marcus A. Mall, Petra Knaup, Mark O. Wielpütz, Urs Eisenmann

**Affiliations:** ^1^Institute of Medical Informatics, Heidelberg University, Heidelberg, Germany; ^2^Translational Lung Research Center Heidelberg (TLRC), German Center for Lung Research (DZL), Heidelberg, Germany; ^3^Department of Diagnostic and Interventional Radiology, University Hospital Heidelberg, Heidelberg, Germany; ^4^Department of Diagnostic and Interventional Radiology with Nuclear Medicine, Thoraxklinik at University Hospital Heidelberg, Heidelberg, Germany; ^5^Department of Pediatric Respiratory Medicine, Immunology and Critical Care Medicine, Charité-Universitätsmedizin Berlin, Berlin, Germany; ^6^German Center for Lung Research (DZL), Associated Partner Site, Berlin, Germany; ^7^Berlin Institute of Health (BIH) at Charité-Universitätsmedizin Berlin, Berlin, Germany; ^8^Division of Pediatric Pulmonology & Allergy and Cystic Fibrosis Center, Department of Pediatrics, University Hospital Heidelberg, Heidelberg, Germany; ^9^Department of Translational Pulmonology, University Hospital Heidelberg, Heidelberg, Germany

**Keywords:** deep learning, magnetic resonance imaging, cystic fibrosis, lung segmentation, pediatric

## Abstract

**Introduction:**

Segmentation of lung structures in medical imaging is crucial for the application of automated post-processing steps on lung diseases like cystic fibrosis (CF). Recently, machine learning methods, particularly neural networks, have demonstrated remarkable improvements, often outperforming conventional segmentation methods. Nonetheless, challenges still remain when attempting to segment various imaging modalities and diseases, especially when the visual characteristics of pathologic findings significantly deviate from healthy tissue.

**Methods:**

Our study focuses on imaging of pediatric CF patients [mean age, standard deviation (7.50 ± 4.6)], utilizing deep learning-based methods for automated lung segmentation from chest magnetic resonance imaging (MRI). A total of 165 standardized annual surveillance MRI scans from 84 patients with CF were segmented using the nnU-Net framework. Patient cases represented a range of disease severities and ages. The nnU-Net was trained and evaluated on three MRI sequences (BLADE, VIBE, and HASTE), which are highly relevant for the evaluation of CF induced lung changes. We utilized 40 cases for training per sequence, and tested with 15 cases per sequence, using the Sørensen-Dice-Score, Pearson’s correlation coefficient (*r*), a segmentation questionnaire, and slice-based analysis.

**Results:**

The results demonstrated a high level of segmentation performance across all sequences, with only minor differences observed in the mean Dice coefficient: BLADE (0.96 ± 0.05), VIBE (0.96 ± 0.04), and HASTE (0.95 ± 0.05). Additionally, the segmentation quality was consistent across different disease severities, patient ages, and sizes. Manual evaluation identified specific challenges, such as incomplete segmentations near the diaphragm and dorsal regions. Validation on a separate, external dataset of nine toddlers (2–24 months) demonstrated generalizability of the trained model achieving a Dice coefficient of 0.85 ± 0.03.

**Discussion and conclusion:**

Overall, our study demonstrates the feasibility and effectiveness of using nnU-Net for automated segmentation of lung halves in pediatric CF patients, showing promising directions for advanced image analysis techniques to assist in clinical decision-making and monitoring of CF lung disease progression. Despite these achievements, further improvements are needed to address specific segmentation challenges and enhance generalizability.

## 1 Introduction

Cystic fibrosis (CF) is an inherited multi-organ disease, which largely effects the lungs. Repeated bacterial infections and inflammation can result in lung damage, causing most of the morbidity and mortality seen in CF (1, 2). The early detection and monitoring of CF-related lung disease is a prerequisite for optimized care and improved long-term outcomes (3–7).

Recently, chest magnetic resonance imaging (MRI), a radiation-free modality, has shown great promise in assessing structural and functional CF lung abnormalities. Studies have shown chest MRI can detect changes as early as in infancy, and is capable of monitoring disease progression and therapeutic response throughout adulthood (7–14).

To semi-quantitatively assess the severity of lung abnormalities in CF patients, a morpho-functional chest MRI scoring system, also referred to as the Eichinger Score, was developed in 2012 (15). This scoring system includes items for morphological lung abnormalities, as well as perfusion abnormalities (8–11, 15, 16). To automate this scoring process, a critical step is automating the lung segmentation process.

In medical imaging, segmentation refers to identifying an organ or specific tissue of interest by extracting the boundaries and the inner region. This process allows for downstream analysis and extracting important quantitative information within that region. Precise segmentation may support accurate decisions on diagnosis, treatment plans, disease monitoring, and guiding of interventions (17). In the last decade, automated segmentation methods improved in performance and precision, resulting in the possibility of fully automated segmentation in different medical disciplines and imaging modalities (18). Machine learning methods, particularly neural networks, have demonstrated remarkable performance, often outperforming conventional methods, especially when analyzing large datasets (19–21). The nnU-Net, an advanced deep learning framework tailored for medical applications, stands out in its performance (22). It permits the training of networks to perform semantic segmentation with high accuracy and performance, eliminating the need for numerous configuration steps due to its self-configuring training parameters and layer settings. However, difficulties arise when attempting to adapt the nnU-Net to a variety of imaging modalities and diseases. This is particularly challenging when the visual characteristics of pathologic findings deviate significantly from healthy tissue, indicating a change in tissue composition within the same organ (23).

Automated lung segmentation in MR images, especially in the CF population, also have inherent challenges. In MRI, difficulties arise due to the limited spatial resolution and the low contrast between the lungs and the adjacent tissue. In CF patients, breathing artifacts, most notably in young children; cardiac pulsation artifacts; chest growth in children, lung abnormalities displacing air contents, and the deformation associated with disease progression, all contribute to the complexity of the segmentation task (8, 24).

Despite these challenges, many studies are beginning to show promising results incorporating neural networks to automate MRI lung segmentation, even in different underlying pathologies, replacing conventional segmentation approaches (25, 26). Zha et al. applied convolutional neural networks (CNNs) on 3D radial ultra-short echo-times (UTE) oxygen-enhanced MRI in a dataset of 45 subjects (age 10+ with CF, asthma, or healthy) and achieved Dice coefficients of 0.97 and 0.96 for the right and left lung, respectively (27). Furthermore, researchers tested other MRI sequences, such as fast UTE with stack-of-spirals trajectory and matrix pencil decomposition MRI, in CF patients (age 5+) yielding Dice coefficients of 0.96 for children and 0.89 for adults (28, 29).

Notably, Astley et al. tested 2D and 3D nnU-Nets for lung segmentation of patients with varying pulmonary pathologies. In their patient cohort (median age 34 yrs.), analysis of a dataset comprising 809 spoiled-gradient-recalled and UTE MRI scans, even across different vendors, demonstrated a remarkable performance, reaching a median Dice coefficient of 0.96 internally and 0.97 on an external test set (30). To enhance the accuracy of automated lung segmentation, by inclusion of artificially generated images with consolidations, Cristoso et al. reached a Dice coefficient of 0.94 on a cohort of healthy volunteers and patients (31). In a 2023 study of neonates, either healthy or suffering from bronchopulmonary dysplasia, the authors employed CNNs for lung segmentation on quiet-breathing MRI and achieved a Dice coefficient of 0.908 on an internal test set and 0.88 on an independent test set (32). Most recently, a new approach for lung segmentation on healthy adults using thresholding and clustering on an enhanced deep-inspiration-breath-hold reached a Dice coefficient of 0.94 (33, 34). A high benchmark for lung lobe segmentation using pseudo-MRI images derived from CT and three concatenated CNNs achieved a Dice coefficient of 0.95 on a dataset of 100 CF patients over the age of 4.7 years old (35).

To the best of our knowledge, we are the first to demonstrate pediatric lung half segmentations for patients across the entire pediatric age range with different stages of cystic fibrosis using chest MRI on the commonly used sequences BLADE, VIBE, and HASTE. We selected a total of 165 MRI examinations from 84 patients in our internal monocentric CF database. This database contains 1,312 highly standardized annual surveillance MRIs, acquired over more than a decade from 266 patients. Segmentations were created manually by three observers.

## 2 Materials and methods

### 2.1 Study population

This ongoing prospective longitudinal observational study (clinicaltrials.gov identifiers NCT00760071, NCT02270476) was approved by the institutional ethics committee and informed written consent was obtained from the parents or legal guardians of all patients. The CF diagnosis was confirmed by increased sweat chloride (Cl-) concentrations (≥60 mmoL/L) and cystic fibrosis transmembrane conductance regulator (CFTR) mutation analysis. In pancreatic-sufficient patients with borderline sweat test results (sweat Cl- 30–60 mmoL/L), the diagnosis was further supported by assessing CFTR function in rectal biopsies, as previously described (36). We included 165 cases in the study. Some patients were included in our previous reports on morpho-functional MRI (8, 37–39).

### 2.2 Magnetic resonance imaging

We performed standardized chest MRI after the initial CF diagnosis or after referral to our center as early as at the age of 3 months. We repeated exams annually using two 1.5 T scanner models from the same manufacturer (Magnetom Symphony and Magnetom Avanto, Siemens Healthcare, Erlangen, Germany). We kept the scanning protocol constant during the study period, apart from minor updates to new software versions as previously described (8–16, 24). We acquired T1-weighted sequences before and after intravenous application of contrast material and T2-weighted sequences before contrast. Children aged 5 years and younger were routinely sedated with oral or rectal chloral hydrate (100 mg/kg body weight, maximum dose of 2 g).

### 2.3 Staging CF lung disease

One observer (MOW) with more than 15 years of experience in chest MRI, who also evaluated all previous studies, assessed all MRI examinations using the established chest MRI scoring system (8–11, 13–15, 40). The MRI scoring system assigns a numerical disease severity score to each lobe (e.g., 0 = no presence, 1 = <50% of a lobe affected, and 2 = ≥50% of a lobe affected) for each of the morphological score items bronchiectasis/wall thickening, mucus plugging, sacculation/abscess, consolidation, and special finding/pleural lesion, as well as for perfusion abnormalities. The sum of morphological findings becomes the MRI morphology score, perfusion abnormalities create the MRI perfusion score, and the sum of both results in the MRI global score, ranging from 0 to 72.

### 2.4 Image sequence selection

Three MRI sequences in coronal orientation were used (Table 1):

1. Balanced Steady State Free Precession Line Acquisition with Undersampling (BLADE): This is a T2-weighted turbo spin echo-based 2D sequence designed to reduce motion artifacts in MRI. It is particularly useful for imaging areas of the body that are prone to movement, like the lungs, or for imaging patients who have difficulty remaining still (41). Its acquisition can be split among multiple breath-holds (i.e., slices are not necessarily at the same depth of inspiration) or triggered using a navigator signal.
2. Volumetric Interpolated Breath-Hold Examination (VIBE): This is a T1-weighted 3D gradient echo sequence acquired after injection of a contrast agent. It was acquired in a single breath-hold and allows for high spatial resolution (42).
3. Half-Fourier Acquisition Single-Shot Turbo Spin-Echo (HASTE): This is a T2-weighted turbo spin echo 2D sequence that acquires each slice from a single echo train, minimizing motion effects at the cost of noticeable blurring in the phase encoding direction (43).

**Table 1:** MRI sequence details.

	BLADE	VIBE	HASTE
Slice thickness (mm)	4	4	6
Pixel spacing in plane (mm) (min-max)	0.9375*0.9375 - 1.25*1.25	0.78125*0.78125 - 0.879*0.879	0.839*0.839 - 1.875*1.875
				Matrix (min-max)	320*320 - 384*384	512*512	512*512

Since image acquisition protocols changed slightly over the years, pixel spacing and matrix size have different min/max values.

### 2.5 Dataset composition

**Figure 1:**
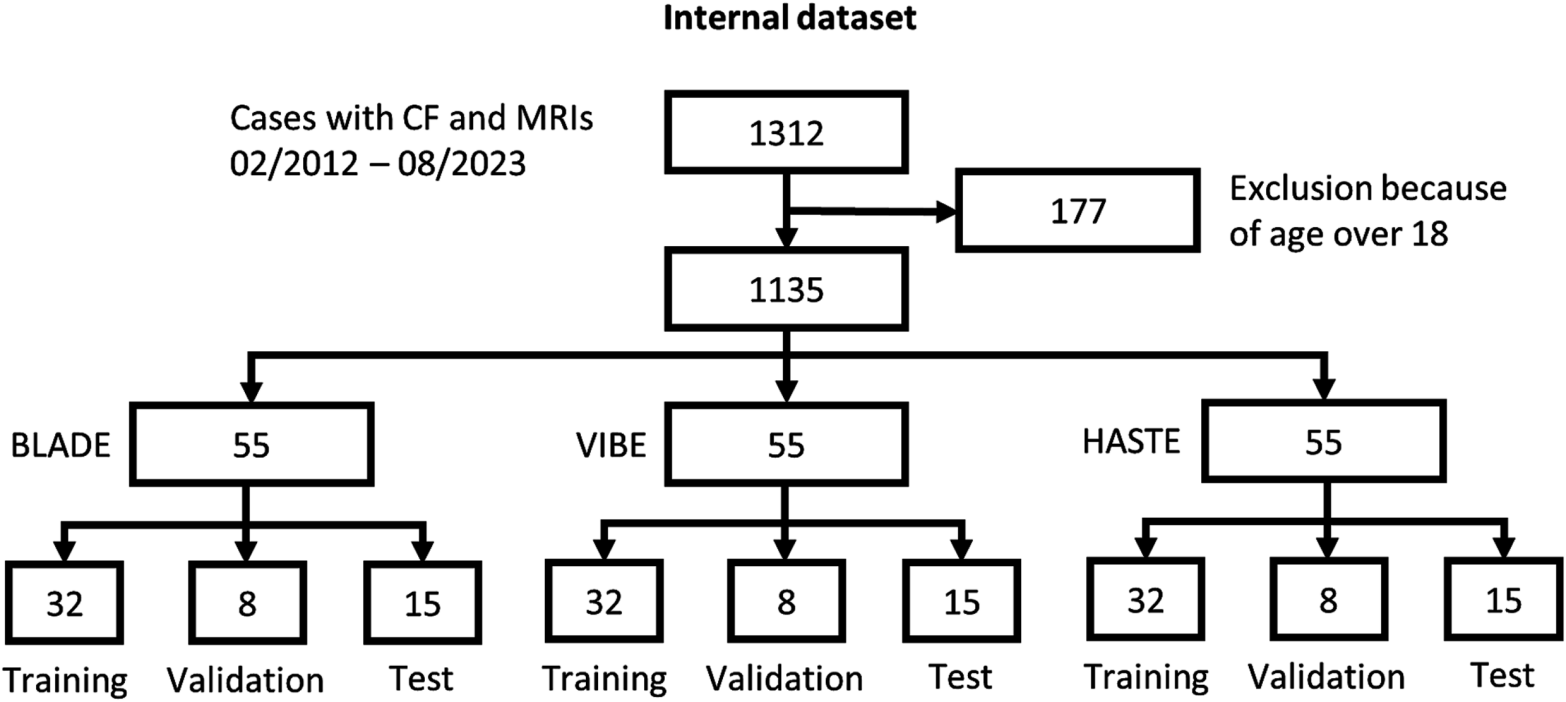
Patient selection flowchart internal dataset.

**Figure 2:**
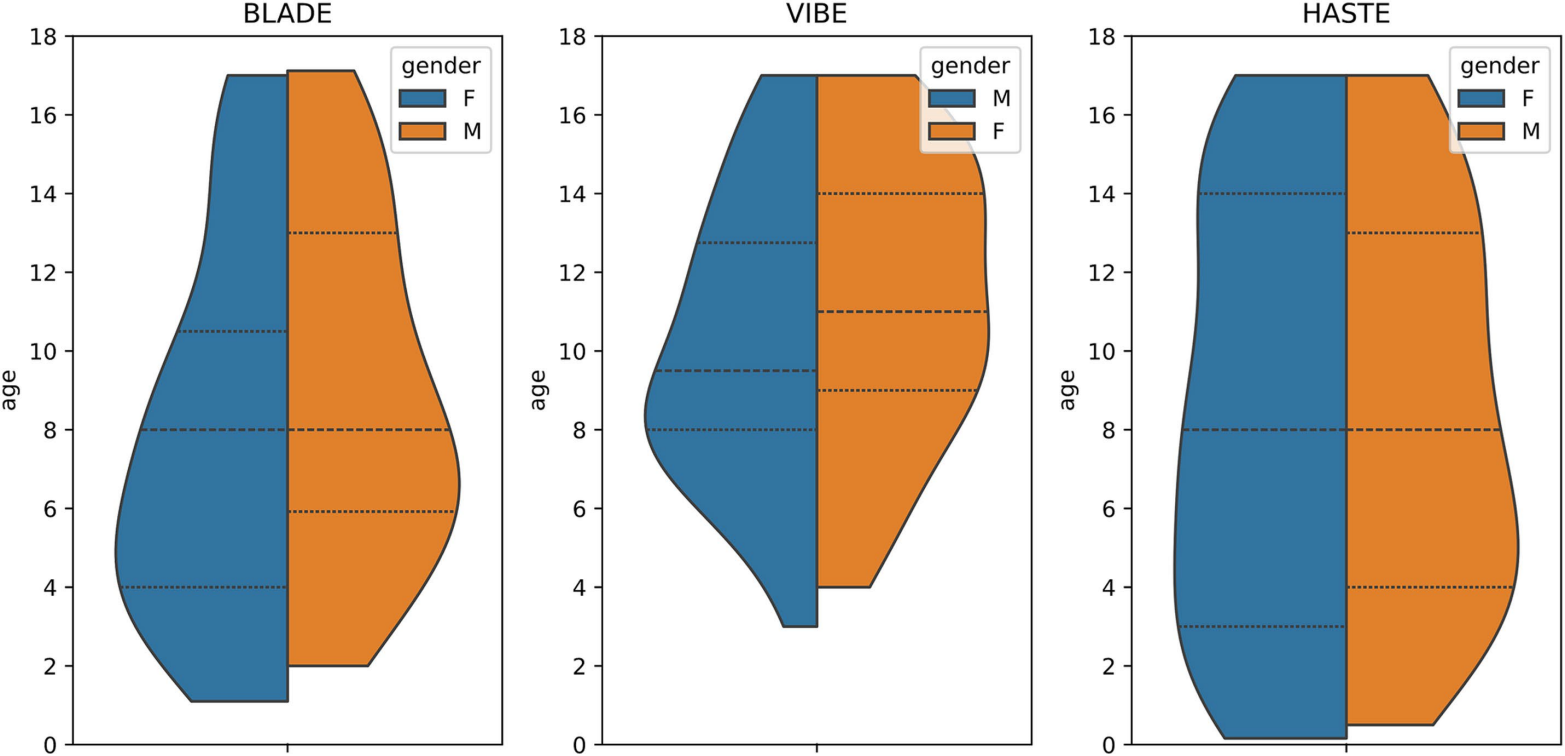
Violin plot of age and gender distribution for the three MRI sequences.

From our database with 1,312 CF examinations from 266 patients, we selected 55 examinations for each MRI sequence (BLADE, VIBE, and HASTE), resulting in an overall 165 examinations from 84 patients (Figure 1). All cases were chosen to ensure an even distribution of age and gender, and to include varying levels of disease severity based on the global MRI score. To achieve this, the overall distribution of age, gender and disease severity was visualized and cases were then selected manually. From this overall dataset with 165 cases, 45 cases (15 for each sequence) were selected in a stratified manner, to represent the underlying distribution of age, gender, and global MRI score for the creation of the internal test set. This internal test set was not used for training, and solely utilized to test the final performance of the networks. In the internal test set, the median age was 9 years (± 4.92) (range 2 months–17 years) (Table 2, internal test set) with 46.7% male cases. The remaining cases were used for training the neural networks in the so-called training set. The training set had a median age of 9 years (± 4.78) (range 2 months–17 years) and 49.1% male cases (Table 3; Figures 1, 2). Selecting the cases in such a way may allow good segmentations over all age and disease classes. We included cases only if relevant image data were available. No cases were excluded due to artifacts or poor quality. With regard to similarity, no notable differences were observed in the global MRI score across the three utilized sequences (*p* = 0.78). A notable difference in age was observed between the three sequences (*p* = 0.006). Patients undergoing imaging using HASTE were notably younger, as HASTE is a contrast agent-free alternative to VIBE. The comparison between the internal training and test data revealed no statistically significant changes in either age (*p* = 0.06) or global MRI score (*p* = 0.97). The available data from all three sequences resulted in 6,010 2D slices. The training set comprised a total of 4,290 slices, with an average of 34 slices for BLADE, 49 slices for VIBE and 24 slices for HASTE per MRI. In the internal test set, a total of 1,720 slices were used, with an average of 36 slices for BLADE, 52 slices for VIBE, and 27 slices for HASTE.

**Table 2:** Overview of the internal and external test sets.

	Internal test set	External test set
Cases, *n*	45	9
Age (years) median [range] (std)	9 [0.13–17.1] ± 4.92	0.79 [0.16–2.0] ± 0.63
Sex (m/f)	21 / 24	4/5
Height (cm) median [range] (std)	133.45 [93.0–174.8] ± 24.43	75.16 [62.0–83.0] ± 7.73
Weight (kg) median [range] (std)	28.03 [12.4–55.9] ± 14.65	9.2 [6.0–10.6] ± 1.73
CF*TR* genotype, *n* (%)	38 (84)	4 (44)
*F508del/F508del*	18 (40)	2 (22)
*F508del/other*	15 (33)	3 (33)
*Other/other*	4 (9)	
Pancreatic insufficiency, *n* (%)	31 (67)	9 (100)
Spirometry, *n* (%)	29 (65)	0 (0)
*ppFEV1* median [range] (std)	97.7 [62.2–123.7] ± 15.8	-
Multiple breath washout, *n* (%)	26 (58)	6 (67)
*LCI N_2_* median [range] (std)	7.74 [4.74–10.57] ± 1.4	7.8 [6.58–9.05] ± 1.2
Global MRI Score median [range] (std)	12 [0–43] ± 9.8	10.22 [7–16] ± 2.72

**Table 3:** Patient characteristics of the internal dataset (training set).

	Internal training set
Cases, *n*	120
Age (years) median [range] (std)	9 [0.16–17.0] ± 4.78
Sex (m/f)	59/61
Height (cm) median [range] (std)	135 [52.7–175.3] ± 24.80
Weight (kg) median [range] (std)	28.2 [12.8–70.9] ± 16.05
CF*TR* genotype, *n* (%)	108 (90)
*F508del/F508del*	44 (37)
*F508del/other*	48 (40)
*Other/other*	12 (10)
Pancreatic insufficiency, *n* (%)	90 (75)
Spirometry, *n* (%)	46 (38)
*ppFEV1* median [range] (std)	91.58 [42.8–108.5] ± 16.3
Multiple breath washout, *n* (%)	45 (37.5)
*LCI N_2_* median [range] (std)	8.28 [3.91–15.3] ± 2.46
Global MRI score median [range] (std)	12 [0–39] ± 7.83

Additionally, we collected an external dataset from two different centers (Center A: one case, Center B: eight cases) comprising nine HASTE acquisitions from nine cases (Table 2) (44). Compared to the internal dataset, the age distribution of the external test set was dominated by very young patients (Table 2). This was reflected in the statistically significant difference in age between the internal and external datasets (*p* < 0.0005). Regarding disease severity, however, the external patients were similar to those of the internal dataset. A comparison of the global MRI score exhibited no notable differences (*p* = 0.86). The external test set had an overall number of 174 2D slices with an average of 19 slices per MRI.

### 2.6 Segmentation ground truth generation

We manually segmented all MRIs using three independent observers with 1 (JM), 2.5 (FGR), and 5 years (LW) experience in lung MRI segmentation, respectively. They created reference segmentations of the lung halves using the open-source software Medical Imaging Interaction Toolkit (MITK, version 2021.10) in combination with a Wacom Cintiq 16 tablet and pen. In the event of disagreement among observers, agreement was reached by individual comparison, collective discussion and consensus among the observers.

### 2.7 Segmentation questionnaire

In cooperation with the experienced radiologists, we designed a qualitative questionnaire for fine-grained evaluation of the segmentations (Supplementary Figure 1). The questionnaire evaluated the overall segmentation quality on an 11-point Likert-scale (45), ranging from 0 (worst quality) to 10 (best quality). Furthermore, the questionnaire included a detailed evaluation of the lung segmentation and specific information regarding the segmentation performance in specific anatomical regions (ventrally, dorsally, mediastinum, periphery, apex, and diaphragm). Information on incomplete segmentation or over-segmentation in specific areas could be provided. Lastly, the observer was given the opportunity to provide an open text response to the segmentation.

### 2.8 Slice based qualitative analysis

To gain further insight into the segmentation quality, all lungs from the internal test set were subjected to a detailed examination by a radiologist to identify any instances of incorrect segmentation. For each lung, the number of slices requiring correction was annotated. In conjunction with the data on the overall slices, this provides an indication of the quantity of usable slices. The number of slices requiring correction is reported as a mean percentage, with standard deviation and maximum.

### 2.9 nnU-Net implementation

The latest implementation of the 2D nnU-Net (Version 2) was utilized in its default configuration. It is a self-configuring framework, which automatically adapts its architecture, pre-processing, and training pipeline to a given dataset. The nnU-Net framework employs a U-Net-based architecture comprising an encoder-decoder structure. On the encoder path, the spatial dimensions of the input image are successively reduced through convolutional layers and max-pooling, thereby capturing increasingly abstract feature representations. On the decoder path, upsampling is applied to restore the spatial dimensions, concatenating feature maps from the corresponding encoder layers. This allows for high-level semantic information and precise localization. For further details to the nnU-Net, please refer to (22). Three individual nnU-Net configurations were trained, one for each sequence using the following steps: based on the 55 study cases per sequence, the data were partitioned into 58% as training set, 14% as validation set, and 27% as test set. This resulted in 32 cases being used for training, eight cases for validation and 15 cases for testing per sequence. The training and validation sets were utilized for the initial and fine-tuning training of the neural network, while the test set was withheld for final evaluation. To ensure an average performance, we validated the models utilizing 5-fold cross-validation with different training and validation set partitions as per default nnU-Net configuration. All calculations were performed on two Tesla V100S PCIe 32GB with 1,000 epochs and an average run time of 33 h per fold. The batch size was subject to variation during the training phase, with values of 14, 32, and 33, respectively, being applied to BLADE, VIBE, and HASTE. Stochastic gradient descent was employed for optimization purposes, with a weight decay of 3e^−5^ and an initial learning rate of 0.01. *Z*-score normalization was utilized as the normalization method. For inference and producing the final predictions the nnU-Net uses an ensemble of all five folds, reporting one final result for the test set. For external validation a separate dataset was utilized. This external dataset was chosen to simulate real-world scenarios and challenges, ensuring a comprehensive examination of the model’s performance across diverse imaging conditions.

### 2.10 Statistical analyses

A one-way ANOVA test was used to determine if there were statistically significant differences in global MRI score and ages among the different datasets. Results were considered significant at *p* < 0.05.

Furthermore, the Sørensen-Dice-Score (DSC), calculated from the spatial overlap between the ground truth segmentation (GT) and predicted segmentation (PS), was utilized to evaluate the entire MRI sequence (46). The DSC ranges from 0 to 1, evaluating the quality of the segmentation indicated by the overlap and is defined as follows:


$$DSC=\frac{2\;|PS\cap GT|}{\left|PS|+|GT\right|}$$

First, the Dice coefficient was calculated between each manual segmentation and predicted mask, and subsequently, the mean value was obtained for the entire stack of slices. This process was conducted for both the right and left lungs, as well as for the combination of both lung halves.

Data were analyzed with Python (Version 3.9) using the package SciPy (Version 1.11.4) (47). The Pearson correlation coefficient, indicating strength of linear relationship, was calculated for the DSC vs. age and DSC vs. the global MRI score (48). In general, the Pearson correlation coefficient measures the linear correlation of two sets of data and is defined as:


$$r=\frac{\sum \left(x_{i}-\bar{x}\right)\left(y_{i}-\bar{y}\right)}{\sqrt{\sum {\left(x_{i}-\bar{x}\right)}^{²}\sum {\left(y_{i}-\bar{y}\right)}^{²}}}$$

Since the Sørensen-Dice-Score does not provide any indications regarding the location of incorrect segmentations or crucial errors, we deployed an additional questionnaire, which was filled out once for each internal case. To assess the generalizability and robustness of lung segmentation, we conducted an evaluation using an external dataset distinct from the training and validation sets. Due to data availability, only the HASTE model was tested.

## 3 Results

### 3.1 Internal and external test set demographics

A total of 45 cases were utilized for the internal test set, while the external test set consisted of nine cases from two distinct centers. The cases from the external dataset are notably younger, with a median age of 0.79 years, whereas the internal test set had a median age of 9 years (Table 2). With regard to the global MRI score, the external dataset exhibited a slightly lower median of 10.22, as compared to the internal test set, which had a median global MRI score of 12.

### 3.2 BLADE, HASTE, and VIBE are equally well suited for nnU-Net training

**Figure 3:**
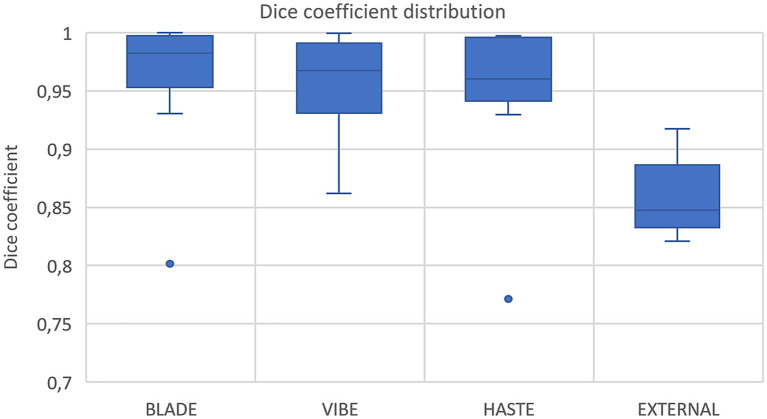
Box plots with dice coefficient of internal test sets from BLADE, VIBE, and HASTE and the external test set.

**Figure 4:**
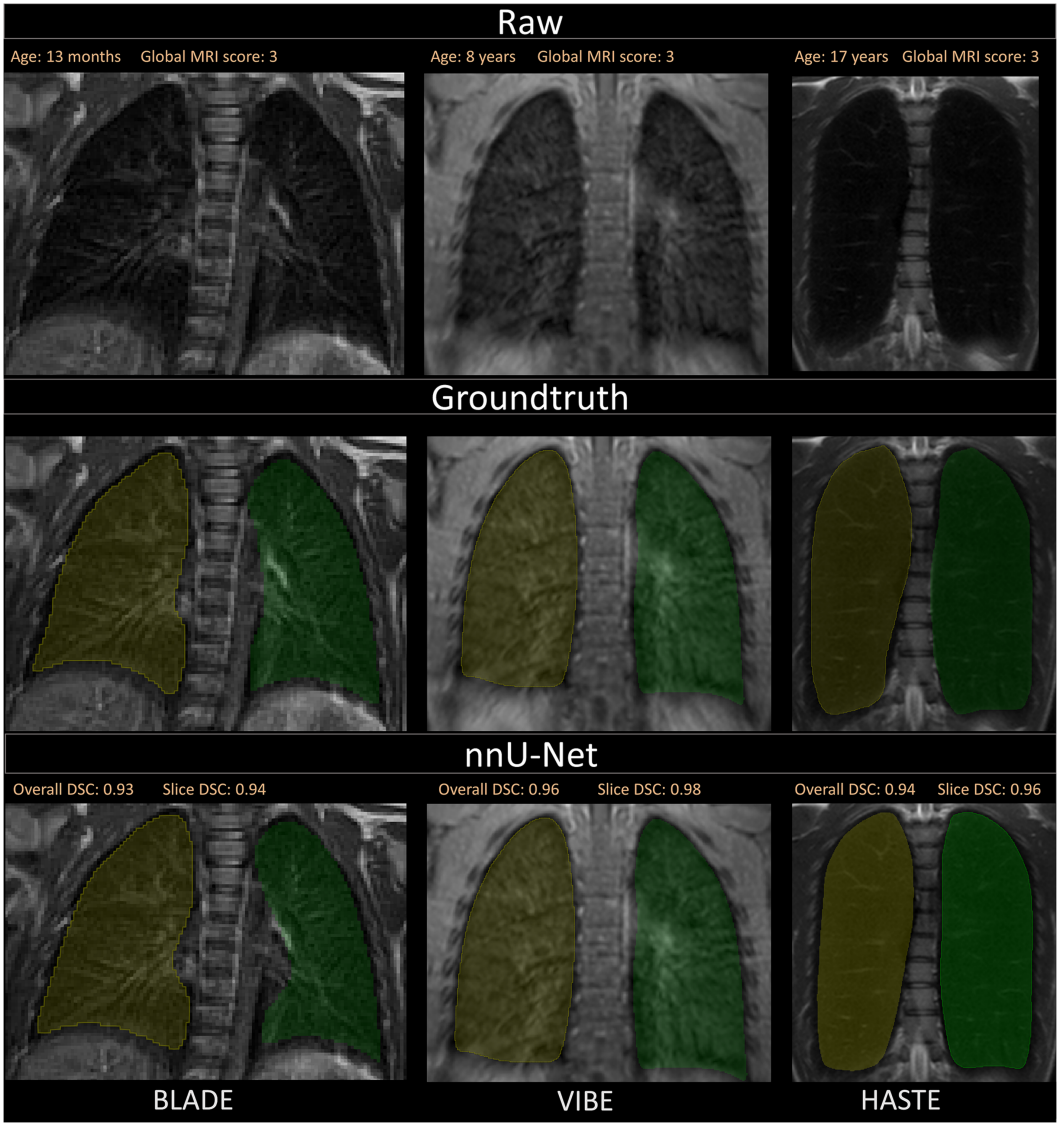
Visualization of the three different sequences with ground truth segmentations and good segmentations produced by the nnU-Net. Overall DSC corresponds to the dice coefficient of the entire lung and Slice DSC to the dice coefficient of the visualized slice. The segmentation of the right lung is indicated in yellow and the left lung segmentation in green. Each column corresponds to one MRI sequence. In the top row, the raw images are shown. The second row contains the manually annotated lung halves (ground truth). In the third row, the segmentation calculated by the corresponding nnU-Net is depicted. The three shown patients are of ascending age from left to right, thus the different lung sizes. All three patients have a global MRI score of 3. The results of the questionnaire indicated that the lungs were rated with a score of 10/10 for BLADE, 9/10 for VIBE, and 9/10 for HASTE. The different contrasts and gray levels are due to the different sequences. Both the ground truth and the segmentation of the lung halves appear to be very similar. Although the right and left lung differ in size and shape, segmentation performance seems to be almost equal. In general, the high Sørensen-Dice-Score and corresponding high segmentation performance are evident.

Using VIBE and BLADE, the nnU-Net achieved a mean DSC of 0.96 (Table 4; Figures 3, 4). HASTE demonstrated comparable performance with a mean DSC of 0.95. For the BLADE sequence, the right lung exhibited slightly superior segmentation, whereas both lungs demonstrated equivalent performance in the VIBE sequence. On the HASTE sequence, the left lung reached a higher DSC compared to the right lung with a DSC of 0.96 and 0.93, respectively (Table 4).

**Table 4:** Sørensen-Dice-Score (DSC) results for three sequences, showing mean (stdv).

DSC	BLADE	VIBE	HASTE
*Whole lung*	0.96 ± 0.05	0.96 ± 0.04	0.95 ± 0.05
*Left lung*	0.95 ± 0.09	0.96 ± 0.04	0.96 ± 0.03
*Right lung*	0.97 ± 0.03	0.96 ± 0.04	0.93 ± 0.10

### 3.3 Questionnaire confirms segmentation quality

**Figure 5:**
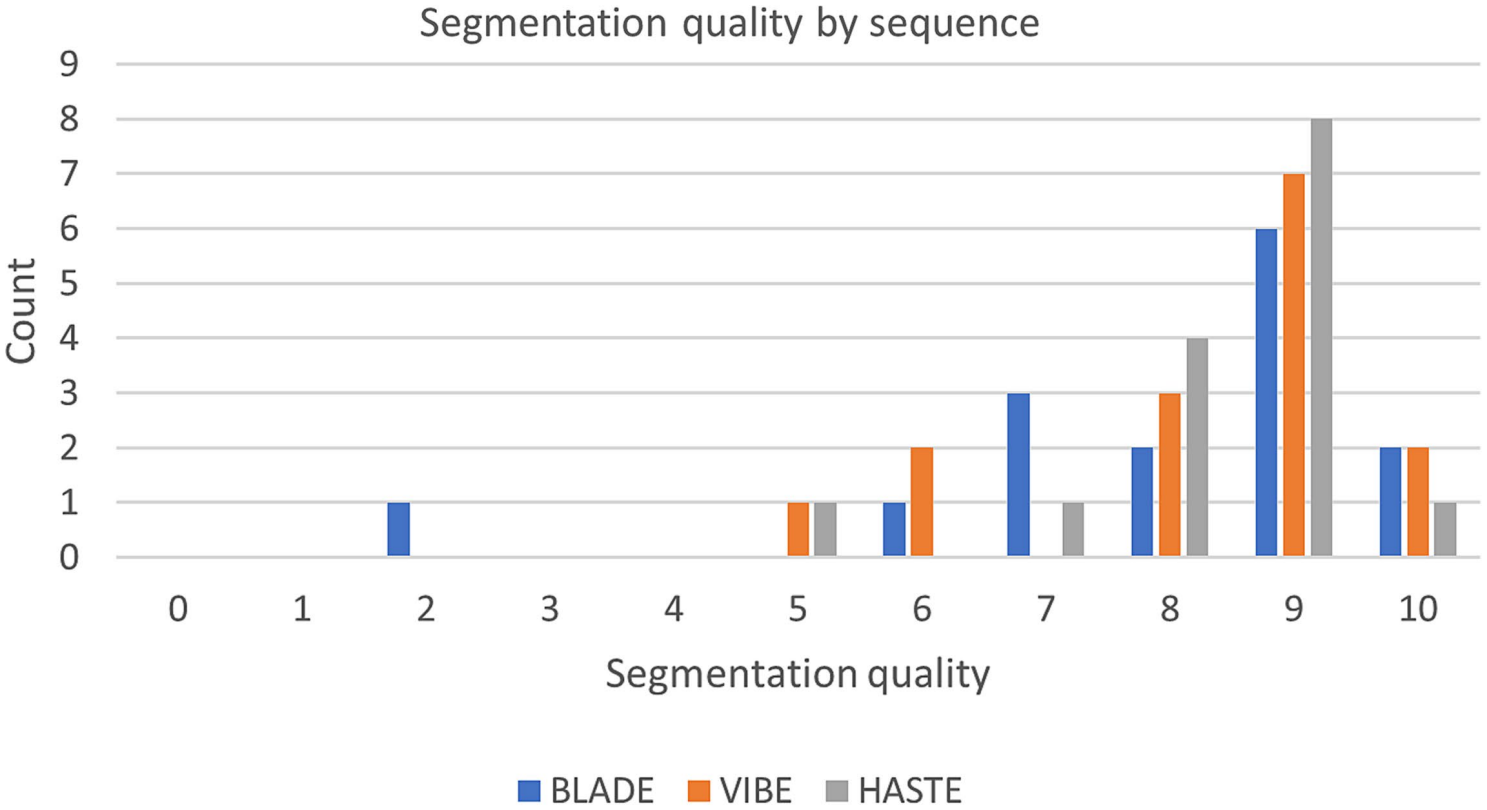
Segmentation quality by sequence with the questionnaire evaluating the segmentations from 0 (bad) to 10 (good) of the internal test set.

Our analysis of the questionnaire for the 45 internal test cases showed similar results to the overall high Dice coefficients. The segmentations derived from all three sequences were evaluated with a median score of nine out of 10 points (9/10) on the Likert Scale, with a standard deviation of 2.02, 1.48, and 1.18 for BLADE, VIBE, and HASTE, respectively (Figure 5). Additional information can be found in Supplementary Figures 2, 3. In addition to the quality of the segmentation, the observer provided information about inconsistencies or errors in the segmentations. Three general trends were identified (Supplementary Figure 3):

1. missing ventral segmentations;
2. missing segmentations near the costodiaphragmatic recess; and
3. incorrect segmentation of the lower mediastinum.

**Figure 6:**
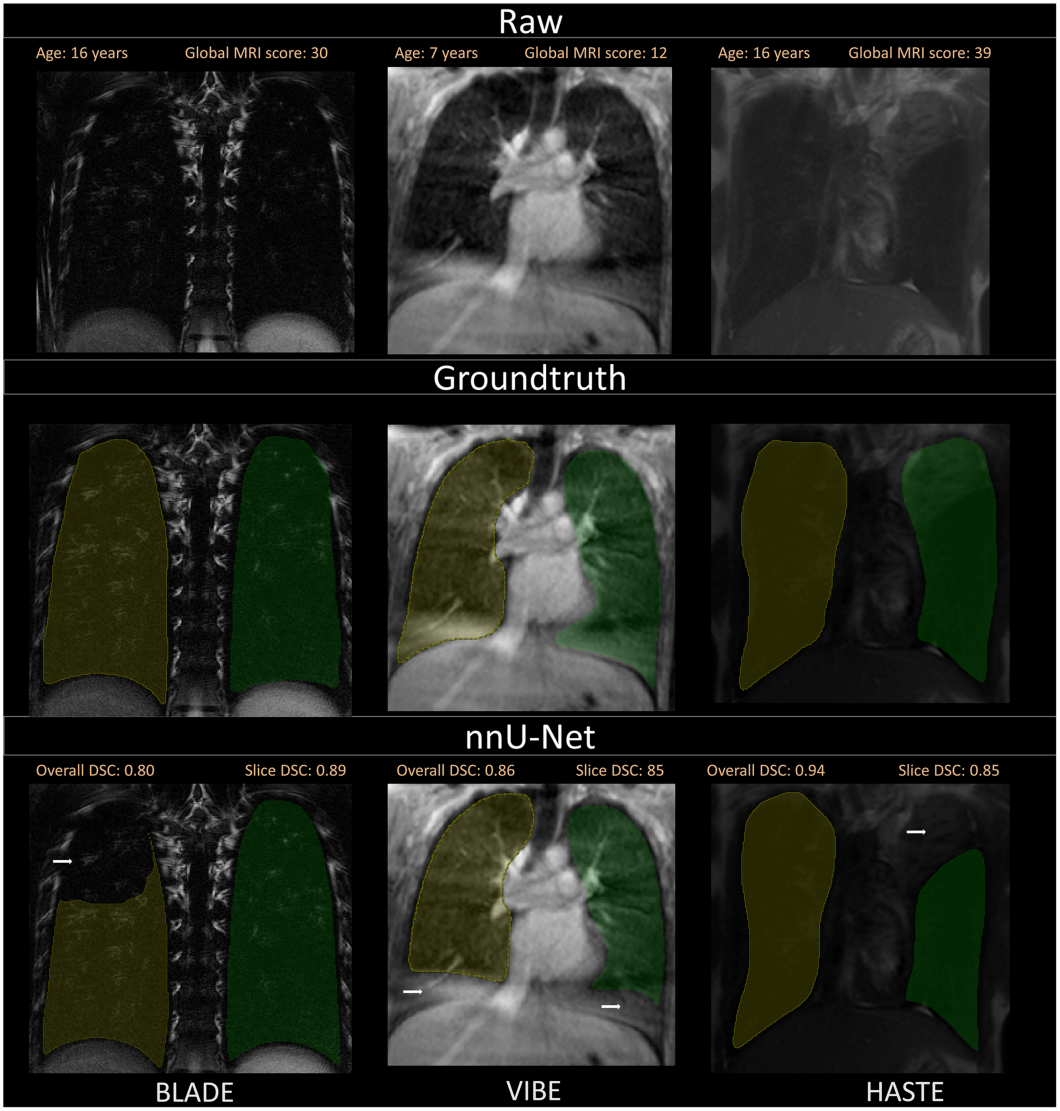
A selection of segmentations with a slightly lower Sørensen-Dice-Score, as well as visual discrepancies between ground truth and nnU-Net segmentation is shown. Overall DSC corresponds to the dice coefficient of the entire lung and Slice DSC to the dice coefficient of the visualized slice. Segmentation errors are indicated with white arrows in the second row. Three common segmentation mistakes are shown: Incomplete segmentations for BLADE, wrong segmentations due to breathing motion or other artifacts for VIBE and pathological changes influencing segmentation performance on the patient captured with the HASTE protocol. Based on the results of the questionnaire, the lungs were rated with a score of 2/10 for BLADE, 6/10 for VIBE, and 7/10 for HASTE.

Further, in some cases, segmentations were incomplete in the lung periphery, leaving a small space unaccounted for close to the edge of the lung (Figure 6).

### 3.4 nnU-Net performance is independent of age and disease severity

Anatomy such as form and size of the chest change with age, and lung disease severity alters anatomy and signal of the lungs. Thus, we correlated DSC with the patient age and DSC with disease severity. Age as well as the MRI global score did not show an association with nnU-Net performance *r* = 0.09 and *r* = −0.12, respectively (Supplementary Figures 4, 5).

### 3.5 nnU-Net shows acceptable performance on external validation data

Nine cases with corresponding HASTE MRI from two external centers, with ages ranging from 3 months to 2 years were segmented using the network trained on the HASTE imaging data. The average DSC across all validated cases was 0.85 (± 0.03) with a range from 0.82 to 0.92 for both lung halves combined (Figure 3, external data). Regarding lung halves, the left lung was segmented better, with a DSC ranging from 0.79 to 0.92, compared to the right lung with a DSC of 0.70 to 0.92. This indicates acceptable, but not perfect performance.

### 3.6 Slice based analysis highlights segmentation quality

A visual inspection was conducted on all data from the internal test set to ascertain the quantity of slices that would require manual correction. A total of 1,720 slices from the 45 internal test cases were subjected to quality control. The results are consistent with the responses provided in question 1 of the questionnaire. The CF case with the lowest score assigned by the radiologist (2/10) exhibited the highest number of slices requiring correction. Specifically, 79% of slices in the right lung and 29% of slices in the left lung were of insufficient quality. Overall, the mean percentage of slices in the right lung and left lung that required correction was 10.60% (±16.46) and 8.75% (±9.39), respectively (Supplementary Figure 6).

## 4 Discussion

Segmentation can play a vital role as a pre-processing step before applying machine learning-based image analysis methods. In our work, lung half segmentation of pediatric MRIs of CF patients using three different sequences, BLADE, VIBE, and HASTE were created utilizing the nnU-Net neural network. A dataset comprising 165 cases, with 55 cases for each of the three sequences, was employed for the training, validation, and testing of the nnU-Net. For each sequence, the nnU-Net was trained individually using a training set of 40 cases and a testing set of 15 cases. For evaluation, the Sørensen-Dice-Score was used in combination with a tailored questionnaire and a slice-based analysis to provide a more detailed insight into the quality of the segmentations.

Overall, the segmentation performance achieved a mean Dice of 0.95 or higher for all sequences and lung halves except for the right lung on the HASTE sequence, which reached a mean Dice of 0.93. With the patient’s age ranging from just a few months to 17 years, the segmentation performance was correlated with age. Generally, it was visible that the segmentation quality stayed constant across all pediatric age classes, further supported by Pearson correlation coefficient *r* = 0.09. Due to the different disease status of the patients, the global MRI score was correlated with the Dice coefficient. Patients with both lower and higher global MRI score were segmented equally well, which is supported by the Pearson correlation coefficient of *r* = −0.12. This demonstrates the excellent performance of the nnU-Net for lung lobe segmentation in pediatric chest MRIs within our cohort. The high mean DSC indicates robust segmentation performance, independent of the underlying pathological changes induced by CF in the pediatric stage. An improvement in segmentation performance might be expected with an increased amount of training data (49). However, in segmentation tasks that require precise ground truth annotations, which are extremely time-intensive to generate, necessary trade-offs must be made.

**Figure 7:**
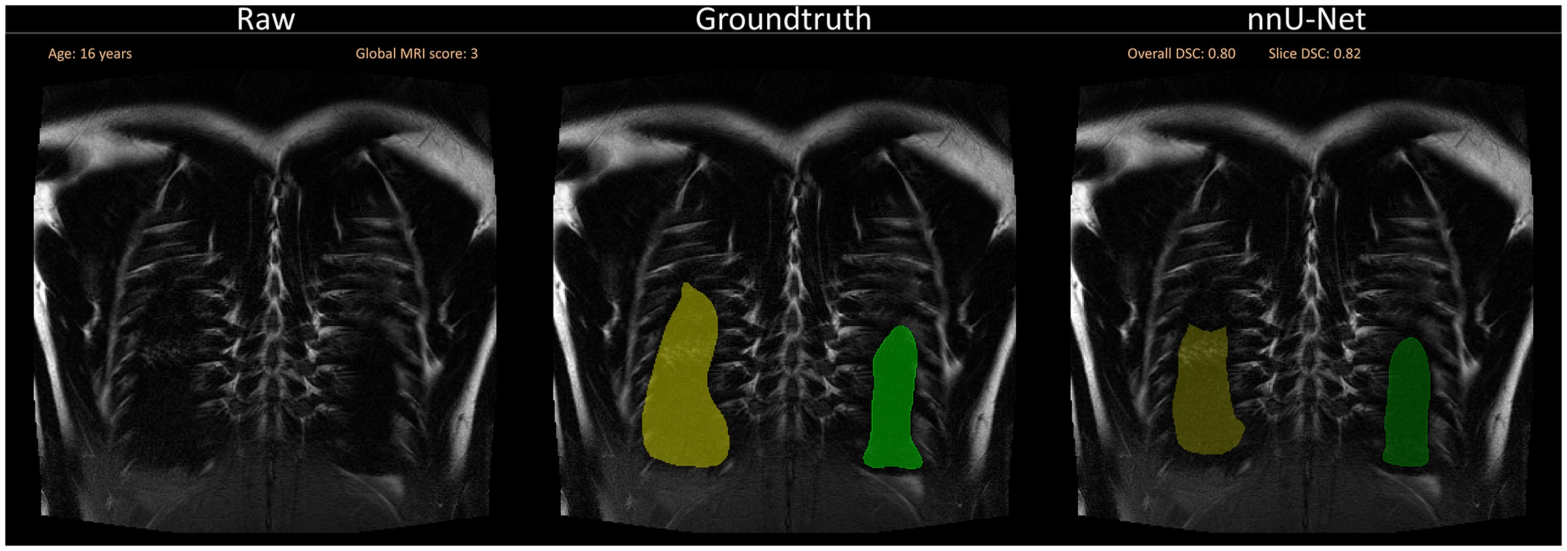
Segmentation errors dorsally due to partial volume effects and general difficulty to segment close to the ribs. The visualization shows a slice close to the back of the patient without any segmentations (left), with the annotated ground truth (middle) and the suggested segmentation by the neural network (right). Overall DSC corresponds to the dice coefficient of the entire lung and Slice DSC to the dice coefficient of the visualized slice.

For a qualitative analysis, we provided a questionnaire to the observers for the purpose of evaluating the segmentations manually in addition to the Sørensen-Dice-Score. Consistent segmentation errors in the ventral and dorsal areas of the lung, as well as around the costodiaphragmatic recess were detected. These errors can be caused by the thickness of the image slices, which directly affects the appearance of the tissue. When the slice thickness increases, tissue other than lung tissue becomes included, which may lead to the partial volume effect (PVE) (50). PVE occurs in volumetric imaging, when more than one tissue type is present in a voxel. In cases where the lung parenchyma ends in the middle of the slice, the voxel will have a different shade of gray compared to voxels completely inside or outside the lung. Depending on the patient and amount of non-lung area on the entire slice, this gray level complicates manual and automated segmentation (Figure 7). Furthermore, even the slightest movement in the ventral and dorsal areas may introduce additional artifacts or blurring. While the observers annotated certain areas as lung tissue, the neural network failed to do so. To overcome this challenge, more annotated data could improve the dorsal and ventral segmentation performance. In addition to the questionnaire, the observer conducted a slice-based analysis annotating which slices required manual correction. The analysis revealed that the left lung necessitated more corrections than the right lung.

To achieve a more general evaluation of the trained neural networks, an external dataset from two other centers was segmented and evaluated with the Sørensen-Dice-Score. This was a useful test to explore whether generalization had been achieved, allowing the processing of data from a different source than the training data. Generally, overfitting on the training data is common, leading to very good performances on the training and test set from the same distribution but poor performance on external data. Despite the fact that the Sørensen-Dice-Score for the external dataset did not exceed 0.92, with a range of 0.7–0.92, it suggests that the trained neural network has overall generalizability, given the differences between the two test sets regarding age and number of slices. A comparison of the number of MRI slices in the internal and external test sets reveals notable differences: the internal dataset averages 38 slices per MRI, while the external dataset averages only 19 slices. Since the Dice coefficient is more sensitive to segmentation errors when the overall segmented area is smaller, this difference in slice count and corresponding segmentation area must be taken into account when interpreting the results (51).

Factors such as different MRI scanner specifications, protocols, slice thickness, and resolution affect image quality and therefore segmentation performance. The small number of external MRIs (*n* = 9) limited the general interpretability. Efforts to improve segmentation performance on the external dataset could include retraining the nnU-Net configuration with external MRI data to reduce segmentation errors. The overall segmentation performances on the three MRI sequences were comparable with existing work in literature. Lung CT scans have been segmented fully automated, reaching high accuracies for lung lobe segmentations with a mean Dice coefficient of up to 0.97 (52). In chest MRI, lung segmentation can be achieved using traditional approaches such as thresholding, but neural network-based segmentation approaches have recently been shown to outperform traditional methods (25). Astley et al. even showed the nnU-Net can be trained to perform well across several sequences, diseases, and vendors reaching a median Dice coefficient of 0.96 on the internal and 0.97 on an independent test set (30). Moreover, their results demonstrated that the 3D-Unet exhibited superior performance compared to the 2D version, which, in turn, outperformed the conventional segmentation approach, spatial fuzzy C-means. In contrast to their work, our study focused on pediatric MRIs of the entire pediatric range of patients with varying degrees of CF disease severity. Efforts toward improving MRI-based lung segmentation include artificially created images to increase robustness in case of severe pathologies (31). For hyperpolarized 129Xe MRI, segmentation performances with a Dice score of 0.929 and above were demonstrated using multiple different methods, highlighting the superiority of the nnU-Net over conventional segmentation methods (53, 54). Neonatal lung segmentations showed a Sørensen-Dice-Score of 0.908 and 0.880 on an independent test set with segmentations automatically by a combination of U-Nets (32).

The resulting segmentations for both the internal and external test sets of the underlying study exhibited variability, yet never attained a Dice coefficient of 1.0. This raises a pivotal question about the criteria for determining whether a segmentation is suitable for subsequent processing or downstream analyses. While a Dice coefficient of 1.0 represents perfect segmentation, striving to improve the coefficient from an already high mean value such as 0.95, may demand a disproportionate amount of time, effort and computational resources. In practice, the pursuit of marginal improvements—bringing the Dice score closer to 1.0—often results in diminishing returns. Such refinements may have minimal impact on the overall effectiveness or accuracy of downstream tasks, particularly when the current segmentation quality is already deemed suitable for clinical decision-making or research purposes. Therefore, it is critical to assess whether the additional time and computational effort invested in further optimizing segmentation is justified, or whether the existing performance is sufficient for the intended applications. When observers segment lungs, they hardly ever reach complete agreement. Segmentation tasks are always dependent on the reader, their experience in the domain, and the tools used. Literature has shown that a Dice coefficient below 0.9 is not uncommon as reader agreement (55). Given that the overarching objective is to automate the Eichinger score, it can be argued that segmentation errors that do not significantly impact the majority of a lung half might be considered acceptable. However, in other research questions, this threshold may have to be set differently, for example, in the context of tumor resection or radiotherapy planning, where segmentations require a higher degree of accuracy (56, 57).

In summary, the results obtained in this study are comparable to those reported in similar studies. To the best of our knowledge, we are the first to demonstrate successful pediatric lung half segmentations for patients with different stages of cystic fibrosis on the MRI sequences BLADE, VIBE, and HASTE.

Our study has some limitations that require discussion. The sample size of 55 cases with corresponding MRIs for each sequence, especially in the age group of patients under 1 year, is relatively small. Compared to the external dataset, all internal cases have larger lungs due to the higher mean age, which could influence the segmentation performance. Moreover, the majority of included patients had a global MRI score of 20 or less. Therefore, it is unclear whether our results are transferable to cohorts of older patients or patients with more advanced lung disease. Future work may focus on this aspect as well as an extension to other sequences.

Recent advancements in this field of research are driving the development of various methods for automated segmentation (19). In the future, it may be valuable to explore these approaches on this dataset and consider expanding the current model to include the remainder of the patients and cases. Especially, since the overall goal of automating the Eichinger score, works toward automated lung lobe segmentations should be explored. Pusterla et al. showed recently that automated lung segmentation with a combination of neural networks is possible with high accuracy (35). Earlier studies demonstrated that segmentation of perfusion maps with a 3D U-Net is an effective approach. However, the evaluation of lung lobes on MRI is challenging due to the difficulty in discerning lobe fissures, if they are visible at all. A lung atlas-based approach, which is independent of age and disease status, may prove advantageous, particularly in light of the findings reported by Tutison et al. regarding the segmentation of the lung (58). With the automated lung segmentation in place, further complex deep learning-based analysis techniques can be applied to assist radiologists in monitoring treatment response, therapy progression, and overall lung health of CF patients, potentially saving time. These results reinforce the efforts toward automated analysis of chest MRIs of patients with cystic fibrosis.

In conclusion, the performance of the nnU-Net in segmenting the lung halves of MRIs from pediatric CF patients demonstrated good agreement with manual segmentations. The segmentation performance of pediatric CF patients does not appear to be significantly influenced by age or disease status.

## Data Availability

The data analyzed in this study are subject to the following licenses/restrictions: the data that support the findings of this work are available from the corresponding author upon reasonable request. Requests to access these datasets should be directed to urs.eisenmann@med.uni-heidelberg.de.
