# Supplementary material for: Automated lung segmentation on chest MRI in children with cystic fibrosis
Source: Front Med (Lausanne). 2024 Nov 12;11:1401473. doi: 10.3389/fmed.2024.1401473 (PMC11600534; doi:10.3389/fmed.2024.1401473)
Supplement: Supplementary file 1 [file Table_1.DOCX]

Supplementary Material

# Supplementary Figures


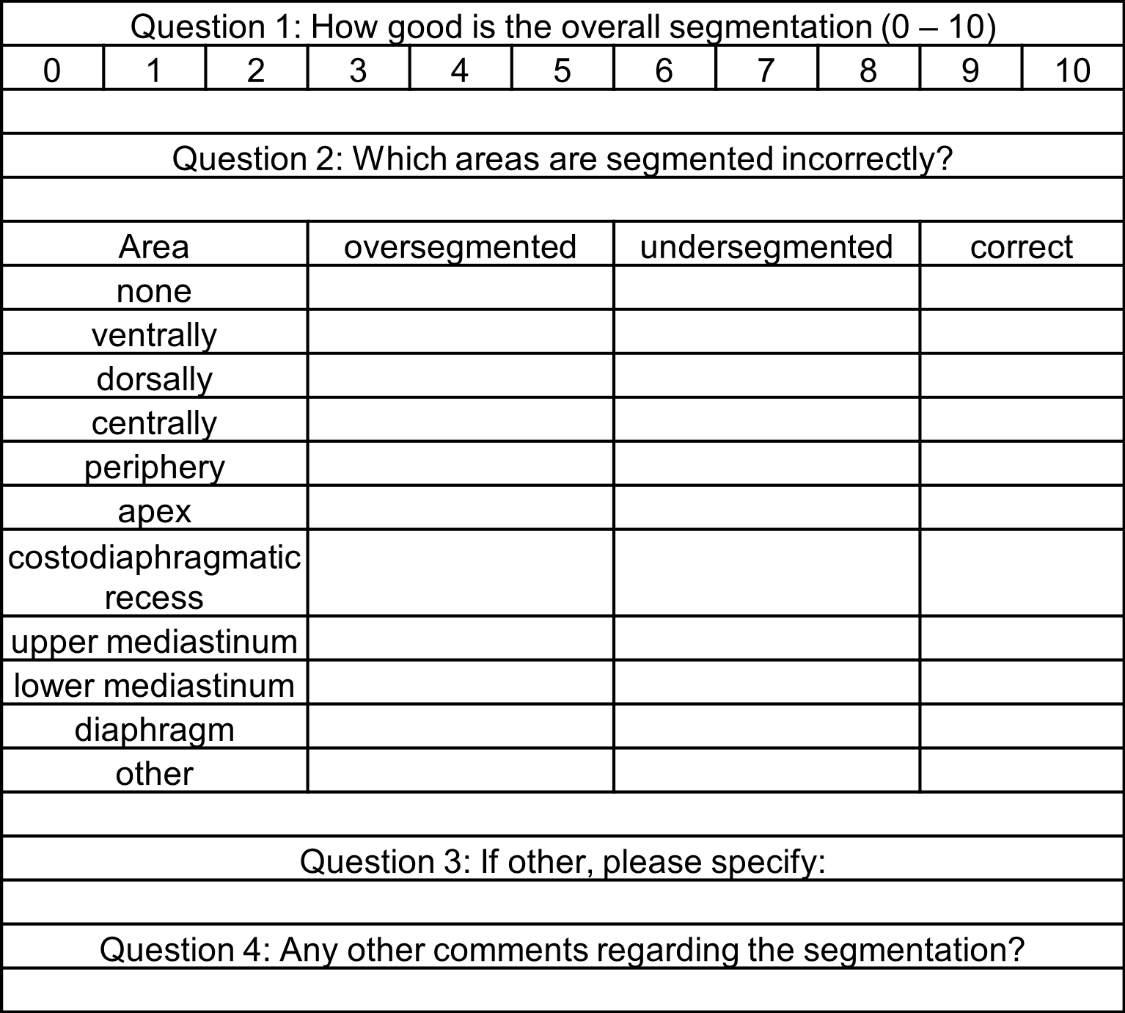


Supplementary Figure 1: Questionnaire used for evaluation of the segmentations.

Supplementary Figure 2: Results from question 1; questionnaire segmentation quality with Likert Scala (0-10).


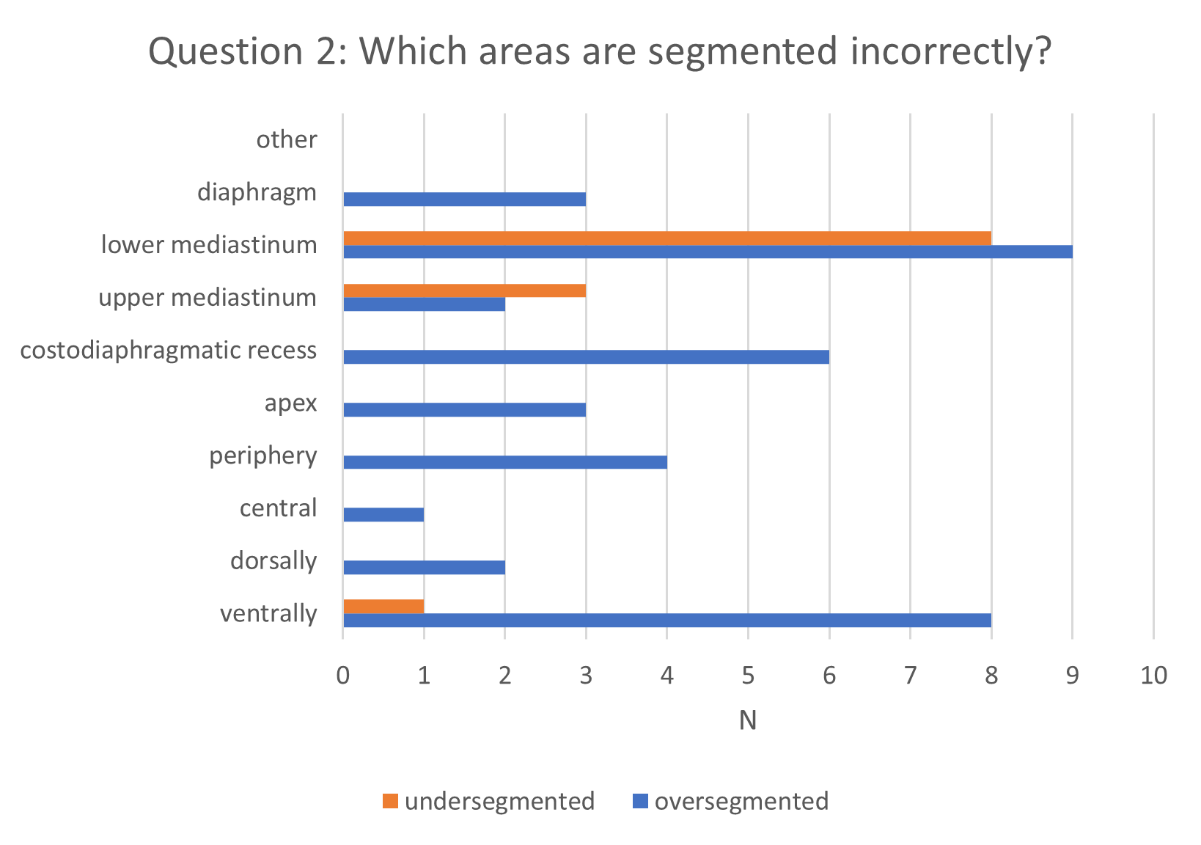


Supplementary Figure 3: Results from question 2; information about over- or undersegmentation in specific areas.

Supplementary Figure 4: Sørensen-Dice-Score (DSC) in correlation with age.

Supplementary Figure 5: Sørensen-Dice-Score in correlation with CF severity in form of the global MRI score.


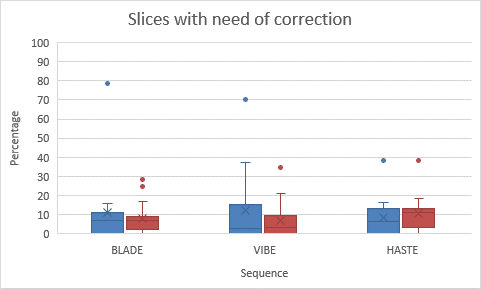

**Supplementary Figure 6:** Boxplot with percentage of slices requiring correction in percentage for each sequence.
